# Spontaneous Renal Artery Dissection in a Patient with Neurofibromatosis Type I

**DOI:** 10.1155/2016/4593932

**Published:** 2016-10-27

**Authors:** Nicolas W. Shammas, Majid Z. Chammas, Jon Robken, Edmund Coyne

**Affiliations:** Midwest Cardiovascular Research Foundation, Davenport, IA, USA

## Abstract

We present a case of spontaneous renal artery dissection (SRAD) in a 28-year-old female with history of neurofibromatosis type I (NF-1) treated successfully with endovascular stenting. The clinical presentation, diagnostic testing, and treatment options are discussed. An endovascular approach with stenting was successfully performed after failure of medical treatment with subcutaneous low molecular weight heparin. Patient's blood pressure and symptoms improved significantly. This may be the first reported case of SRAD in a patient with NF-1 successfully treated with endovascular stenting.

## 1. Introduction

Spontaneous renal artery dissection (SRAD) is a rare complication which is often underdiagnosed and can pose therapeutic challenge. Approximately, 200 cases of SRAD have been documented since the first report in 1944 [1], with 50 of these cases diagnosed at necropsy. In general, renal artery dissections constitute only 1-2% of all arterial dissections [2]. However, isolated SRAD occurs at an incidence of 0.036% to 0.049% of all arterial dissections [3]. The etiology of SRAD is not well defined, but risk factors include hypertension, atherosclerosis, Ehlers-Danlos syndrome, and trauma [2–8].

We report a case of SRAD in a patient with neurofibromatosis type I (NF-1) treated successfully with endovascular stenting. Although stenosis or aneurysm formation has been previously reported in several vascular beds in patients with NF-1 including the renal arteries [9–16], to our knowledge SRAD associated with NF-1 has not been previously reported.

## 2. Case Report

**Figure 1:**
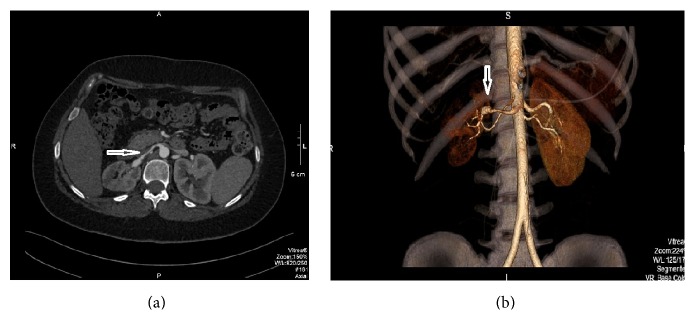
CT angiography of the renal arteries. Transverse view (a) and 3D rendering (b) showing the right renal artery (arrow).

The patient is a 28-year-old female with history of nausea, emesis, and headaches started in December of 2015 with subsequent five emergency visits to a local hospital. She was diagnosed with new onset hypertension with blood pressures running in the 200's systolic and 100's diastolic. She had no associated symptoms of neurologic deficits, chest pain, dyspnea, or heart failure. She described a sudden onset of right flank pain and was evaluated with computed tomography (CT) of the abdomen that showed a large wedge-shaped hypodense areas in the upper pole and posterolateral midpole of the right kidney concerning for acute infarction. A probable thrombus in the right main renal artery was suspected (Figure 1). The patient was treated conservatively with enoxaparin subcutaneous injection 1 mg/kg twice daily and amlodipine for her high blood pressure. She returned to our office February 2016 for a second opinion after persistence of her high blood pressure and nausea. Her blood pressure was 156/100 mmHg and heart rate was 100 beats/min. She had no flank or abdominal tenderness and the rest of her examination was unremarkable. Pheochromocytoma was ruled out with a 24-hour urine collection for metanephrines and catecholamines.

The patient's past medical history is notable for neurofibromatosis type I with fourth ventricle mass status after right-sided ventriculoperitoneal shunt. She does not smoke and has no history of diabetes or hyperlipidemia. She drinks alcohol on occasion. She is single and has no children. Her family history is unremarkable for cardiovascular disease. Given her persistent hypertension and her nausea, renal angiography was advised.

**Figure 2:**
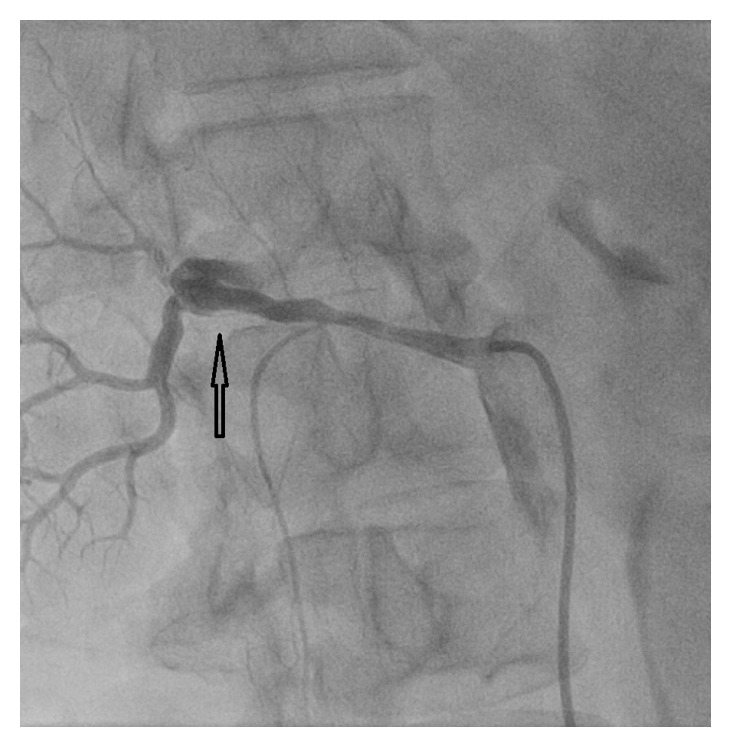
Right renal angiogram showing dissection of the right renal artery (arrow).

After preparation and draping and 1% Xylocaine anesthesia, the right common femoral artery was entered percutaneously via the Seldinger technique and a Hemaquet catheter introducer is advanced into the artery over a guidewire. After abdominal aortography with a pigtail catheter, a Cobra catheter was used to selectively obtain left and right renal angiography and identified the right renal artery dissection (Figure 2).

**Figure 3:**
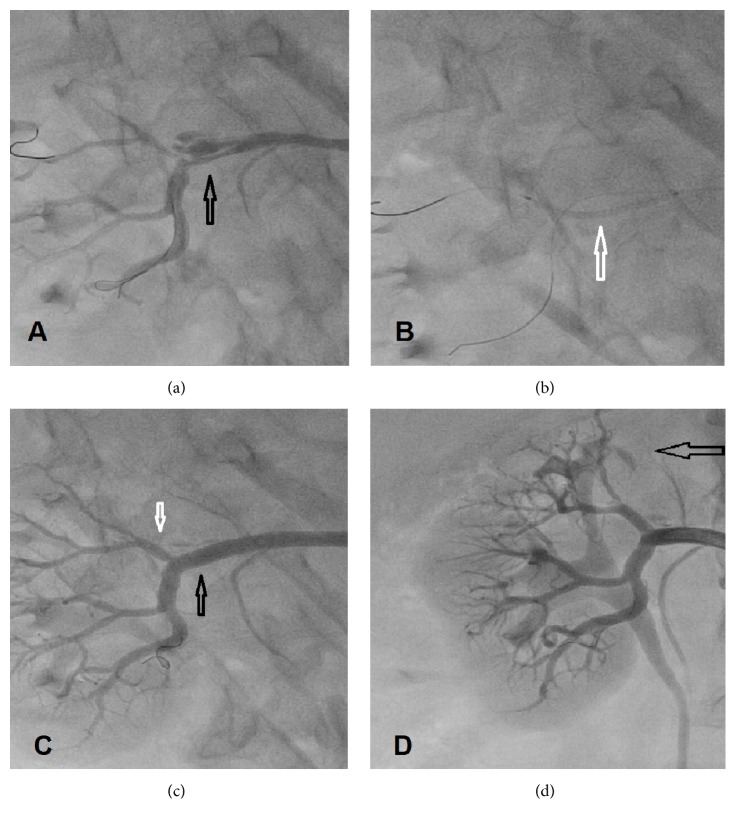
Renal artery treatment. (a) 0.014′′ coronary wire crossing the true lumen (arrow), (b) 2 wires, one in the upper lobar branch and one in the lower branch with balloon dilation of the main trunk into the upper branch (white arrow), (c) stenting of the upper branch (white arrow) and the main branch into the lower branch (black arrow), and (d) final image showing the posttreatment results. Dark open arrow points to the infarcted upper lobe of the right kidney where dye opacification is minimal.

Following the diagnostic angiogram, a renal guiding catheter was then advanced to the right renal artery. Using a turnpike catheter (Vascular Solutions, Inc., Minneapolis, MN), 0.014′′ guidewire access was obtained through the right renal artery into the true lumen of the inferior and superior branches. The upper branch was then dilated with a 2.5 mm Emerge coronary balloon (Boston Scientific, Maple Grove, MN) followed by a 3.0 mm balloon. The superior branch was then stented with a 3 mm by 9 mm Integrity bare metal stent (Medtronic, Minneapolis, MN) deployed at 14 atmospheres of pressure. Stent deployment was then achieved into the inferior branch extending into the main trunk of the right renal artery by using a 4.0 mm × 26 mm Integrity stent which was then postdilated with a 5.0 mm balloon in the main right renal trunk. Kissing balloon was then performed in the superior and inferior branches at 14 atmospheres. The proximal right renal artery was then stented with a 4.0 × 15 mm Integrity stent post dilated by a 5 mm noncompliant balloon (Figure 3). At the end of the procedure, wires and the guiding catheter were removed and the Perclose system (Abbott Vascular, Abbott Park, Illinois) was used to achieve hemostasis in the right common femoral artery. She was placed on oral dual antiplatelet drugs (clopidogrel 75 mg daily) and a baby aspirin (81 mg daily) planned for 6 months then aspirin indefinitely.

Patient was seen in the office 3 months after stenting of her right renal artery. She has kept close records of her blood pressure at home which consistently showed a systolic blood pressure less than 140 systolic. Her systolic blood pressure in the office was 130 mmHg on amlodipine and small dose of hydrochlorothiazide. Her renal function was normal before the procedure (creatinine 0.8 mg per dL) and remained normal 5 months after procedure (0.6 mg per dL). Her symptoms have resolved and she was feeling well.

## 3. Discussion

NF-1, also called von Recklinghausen's disease, is an autosomal dominant genetic disorder linked to chromosome 17 and presents with neural tumors, cutaneous pigmentations, and Lisch nodules. NF-1 is occurs in 1 in 3,000 individuals and has been associated with vasculopathy [9–11]. The renal artery is more frequently involved and presents as renovascular hypertension. Other vascular beds involved in NF-1 include the abdominal aorta, intercostal arteries, subclavian artery, vertebral artery, coronary arteries, and the internal carotid artery with aneurysm formation, dissection, and rupture [9–16]. The mechanisms of vascular involvement include smooth muscle dysplasia and direct vascular invasion by the neurofibromatous or ganglioneuromatous tissue.

Our patient presents with SRAD as a cause of her new onset hypertension. Although renal artery stenosis and aneurysm formation have been reported in patients with NF-1, to our knowledge SRAD has not been previously reported. Our patient presents with flank pain, emesis, and severe hypertension and had demonstrable lobar infarct in her renal artery as a result of the SRAD. She had been managed initially conservatively with low molecular weight heparin and a blood pressure lowering medication. However, her hypertension persisted despite initiating medical treatment. Endovascular treatment of her SRAD was considered and stenting was successfully performed with restoration of good flow to her kidney. On follow-up, her symptoms resolved and blood pressure was controlled on amlodipine and hydrochlorothiazide. This case illustrates that an endovascular approach to treat SRAD is preferred over medical treatment. Patient continued to be symptomatic with nausea and high blood pressure despite 2 months of anticoagulation. Her renal artery also continued to show clear dissection angiographically, which was not very appreciated on her CT scan. Renal angiography yielded superior diagnostic accuracy in this case and is likely to be a more definitive diagnostic method in patients with NF-1 that present with acute flank pain and hypertension. Although the CT suggested a thrombus in the right renal artery, the angiogram was conclusive to the presence of SRAD.

The prognosis of a conservative or endovascular approach in treating NF-1 related SRAD is unknown. Also, it is unclear what would be the long term impact of stenting of the renal artery and whether this would avoid further problems in the future. One of the limitations of this case report is the short follow-up after procedure. We believe this case, however, represents an effective approach in treating SRAD in the setting of NF-1.
